# Development and validation of novel biomarker assays for osteoarthritis

**DOI:** 10.1371/journal.pone.0181334

**Published:** 2017-07-17

**Authors:** Khadija Ourradi, Yunhe Xu, Dominique de Seny, John Kirwan, Ashley Blom, Mohammed Sharif

**Affiliations:** 1 School of Clinical Sciences, University of Bristol, Musculoskeletal Research Unit, Learning and Research Building, Southmead Hospital, Bristol, United Kingdom; 2 Laboratory of Rheumatology, GIGA-I, University of Liege, CHU de Liege, Liege, Belgium; 3 Academic Rheumatology, University of Bristol, The Courtyard, Bristol Royal Infirmary, Bristol, United Kingdom; Consiglio Nazionale delle Ricerche, ITALY

## Abstract

**Background:**

Osteoarthritis (OA) is the most common chronic joint disease usually diagnosed at relatively advanced stages when there is irreparable damage to the joint(s). Recently, we have identified two novel biomarkers C3f and V65 which appear to be OA-specific and therefore potential markers of early disease. We report the development of immunoassays for quantitative measure of these two novel biomarkers.

**Method:**

Monoclonal and polyclonal antibodies were generated by immunising mouse and rabbits respectively with peptide-carrier conjugates of C3f and V65. Affinity purified antibodies were used for immunoassays development and assays validated using serum from OA patients and controls.

**Results:**

The ELISAs developed showed spiked recovery of up to 96% for C3f and V65 peptides depending on serum dilutions with a coefficient of variation (CV) <10%. The intra- and inter-assay CVs for C3f and V65 were 1.3–10.8% and 4.2–10.3% respectively. Both assays were insensitive for measurements of the peptides in patients and the use of different signal amplification systems did not increase assay sensitivity.

**Conclusion:**

We have developed two immunoassays for measurements of C3f and V65 peptides biomarkers discovered by our earlier proteomic study. These assays could detect the endogenous peptides in serum samples from patients and controls but lacked sensitivity for accurate measurements of the peptides in patients. Our study highlights the difficulties and challenges of validating biomarker from proteomic studies and demonstrates how to overcome some of the technical challenges associated with developing immunoassays for small peptides.

## Introduction

Osteoarthritis (OA) is the most common chronic joint disease causing substantial health deficits [1] and is becoming increasingly prevalent as the population ages. By 2020, OA will be the fourth leading cause of disability in the world [2]. Diagnosis of OA depends on patient-reported pain and disability, followed by imaging (usually plain X-ray) and blood biochemistry to rule out other diseases such as rheumatoid arthritis (RA). These tests all concern late-stage disease, and therefore simple, non-invasive biochemical tests that can be used in individual at-risk patients for early diagnosis are urgently needed for more effective management of OA.

Over the last 3 decades, identification of OA-specific biomarker(s) has been the goal of many OA research programmes. Such tests would enable (i) early diagnosis and monitoring OA (ii) provide an improved OA outcome measure in clinical trials and (iii) provide a direct measure of drug effect and mechanism of action to help better tailor personalised medicines for OA treatment. Overall the availability of OA-specific biomarkers should lead to significantly better management of OA and hence reduction in pain and disability for the millions of sufferers in the world. Earlier studies have demonstrated that some serum macromolecules (biomarkers) can provide a way of measuring and monitoring key disease processes such as cartilage loss and bone remodelling in established and advanced disease [3–6].These biomarkers are mostly related to joint tissue turnover and although each has some clear relationship to OA progression in general, all have proven incapable of identifying individual patients in early disease stages and at high-risk progression [7, 8]. In a previous study using mass spectrometry (MS) surface-enhanced laser desorption/ionization–time of flight (SELDI-TOF), we discovered 4 novel biomarkers of OA. The peak intensities of two of these biomarkers showed good discrimination between OA and controls. These two biomarkers were identified as: C3f- a complement fragment released during the catabolic degradation of C3b after C3 complement activation, and V65- a subunit of vitronectin protein, a cell adhesion and spreading factor. Unlike the currently available biomarkers, these markers may reflect cellular metabolism process rather than products of tissue destruction and therefore represent a new generation of more promising biomarkers. Increased serum C3f and V65 appear to be specific for OA patients in comparison to normal control (NC) as well as disease control (RA) and can detect non-radiographic stage of OA (Kellgren & Lawrence (K&L) grade 0), and increases as the radiographic disease severity of OA increases [9]. The aim of the current study is to raise polyclonal and monoclonal antibodies to C3f and V65, develop first generation immunoassays using affinity purified antibodies and carry out validation of the new assays using highly characterised stored serum samples.

## Materials and methods

### Polyclonal and monoclonal antibody production for immunoassays

Monoclonal and polyclonal antibodies were raised by BioServ UK Ltd in Sheffield as a contract research under Home Office Project Licence 40/3371. Polyclonal antibodies were produced by immunising rabbits with peptide-carrier conjugates. A number of different coupling chemistries were used for the preparation of the immunogen. Glutaraldehyde through terminal amino or MBS linkage via a thiol group on an introduced cysteine residue was selected for linking to carrier proteins Keyhole Limpet Haemocyanin (KLH) or Bovine Serum Albumin (BSA). Peptide-KHL was used as the immunogen and peptide-BSA for screening and selection of peptide-specific antibodies. The initial challenge was with Freund’s Complete Adjuvant followed by 2 x boosts utilising Freund’s Incomplete Adjuvant. Immune responses were monitored by taking serum samples and analysing by ELISA. Three rabbits were immunised with each of the peptides but only one in each group responded to the antigens. Once half maximal binding giving a serial dilution of at least 1:10000 were reached, terminal serum samples were obtained and purified using protein G affinity columns. For affinity purification, the peptide was linked via the N-terminal cysteine to a sepharose column matrix using a SulfoLink™ Coupling Resins and Immobilization Kits (Thermo Scientific, 20401, UK) according to the manufacturer’s instruction and the subsequent eluted fractions were tested for specificity on C3f and V65 coated plates by ELISA.

Monoclonal antibodies were produced by hyperimmunising animals with peptide carrier conjugates and assaying for a strong serum response as described above. Some mice did not respond at all to the peptides conjugates. Six animals were immunised for each of the peptides and once good immune response was established in a mouse, the spleen was removed and B cells immortalised by fusing to Sp2 myelomas and hybrids secreting antibody with peptide specificity selected by ELISA. A gamma chain specific anti-mouse enzyme labelled secondary antibody was used in screening all of the hybrids in order to ensure that only monoclonals of high affinity IgG isotypes were selected. A panel of hybridoma lines showing best binding in ELISA assay was cryopreserved and cloned by limiting dilution cloning to single cell homogeneity. For each of the best secreting cell lines, a 1 litre batch of hybridoma cells was produced in roller bottles and antibody purified from this using protein G affinity chromatography. Yields in the order of 20-30mgs/litre was achieved which provided sufficient material for immunoassay development. Finally, monoclonal antibodies to C3f and V65 were selected and affinity purified against free C3f and V65 peptides respectively. We aimed to select a pair of antibody (1 monoclonal and one polyclonal) to each of the peptide for assay development. However, all the monoclonal antibodies produced against V65 showed poor affinity for the peptide and cross-reactivity with unrelated peptides. Therefore, only a polyclonal antibody to V65 was available for immunoassay development.

### Serum sample from patients

Patient and control serum samples used for this study are from the Bristol cohorts used in our proteomic study [9]. Serum samples used were collected and stored under the appropriate ethical approval and International Conference on Harmonisation—Good Clinical Practice (ICH GCP) guidelines as reported in previous studies [7, 10]. The use of these samples for discovery, development and validation of biomarkers for diagnosis and monitoring arthritis was approved by the NHS Health Research Authority NRES Committee, London—Bloomsbury (REC reference number: 14/LO/1046)".

### C3f sandwich ELISA

Sandwich ELISA for detecting C3f was developed by using rabbit polyclonal antibody and monoclonal antibody either as detecting or capturing antibody. The polyclonal/monoclonal combination provided better standard curve and therefore chosen for further studies. Briefly, 96-well plate (NUNC Immunotech, UK) was coated with 100μl/well of rabbit anti-C3f polyclonal antibody at 1μg/ml and incubated overnight at 4C° (Sigma, UK). Wells were then washed with 300μl/well of phosphate buffered saline (PBS) and blocked with 200μl/well of blocking buffer (PBS with 1% BSA (Sigma, UK)) for 1 hour at room temperature (RT). Wells were then washed again prior to addition of 100μl/well of samples and standards (synthetic C3f peptide (Eurogentec, UK)) diluted in assay buffer (PBS with 0.2% BSA and 0.05% Tween-20 (Sigma, UK)) and incubated at 4C° overnight. All samples and standards were applied in duplicates. A nine point standard curve was created using 2-fold serial dilutions with 1μg/ml as the highest standard. Wells were washed 4 times with washing buffer (PBS with 0.05% Tween-20), and 100μl/well of mouse anti-C3f monoclonal antibody at 1μg/ml was added to each well for 2h at RT. An anti-mouse immunoglobulins-biotinylated (Dako, UK) conjugate diluted 1 in 4000 in assay buffer was used at 100μl/well and incubated for 1h to detect the monoclonal antibody. The plate was washed 3 times and incubation with Streptavidin-HRP (horseradish peroxidase) diluted 1/8000 for a further hour. Assay was developed using 100μl/well of OPD (o-phenylenediamine dihydrochloride) substrate (Sigma, UK) and colour development was stopped with 50 μl/well of 1N HCL (Hydrochloric acid) solution (Fisher, UK). Plate was read at 490 nm using a Tecan F50 Absorbance reader (Labtech, UK). A standard curve was generated by nonlinear regression using Graph Pad Prism version 6.05 statistical software (Graph Pad, San Diego, California, USA).

### Spiking and recovery test of C3f in human serum

C3f peptide was added at 1μg/ml into assay buffer as a control (C3f control) and to normal human serum diluted at 1:50, 1:100, 1:200 and 1:400 in assay buffer, and analysed using the ELISA.

### Competitive ELISA for V65

Since only one polyclonal antibody (pAb-V65) was specific for the peptide, we developed a competitive ELISA for detection of V65 peptides. Briefly, 96-well plate was coated with 100μl/well of synthetic V65 peptide (Eurogentec, UK) at 0.5μg/ml and incubated overnight at 4C°. Wells were washed and blocked for 1 hour at RT. Wells were then washed again prior to the addition of standards, consisting of premixed concentrations of synthetic V65 peptide from 0–4μg/ml with polyclonal anti-V65 antibody for 30 minutes in assay buffer. 100μl of the antibody-antigen complexes was then put in duplicate wells and incubated for 1h at RT. Wells were then washed 3 times with washing buffer prior to addition of 100μl of anti-rabbit immunoglobulins-HRP (Dako, UK) conjugate (diluted 1 in 1000) to detect the polyclonal antibody. Assay was developed using OPD substrate. Colour development was stopped with 1N HCL solution and the plate read at 490 nm using a Tecan F50 Absorbance reader (Labtech, UK). For competitive ELISA, the higher the concentration of V65 in the sample, the weaker the optical density (OD) values. A standard curve was generated by nonlinear regression using Graph Pad Prism version 6.05 statistical software.

### Spiking and recovery test of V65 in human serum

2-fold serial dilutions of synthetic V65 peptide from 1μg/ml were applied to normal human serum diluted 1:100 and 1:400 in assay buffer to assess recovery by the competitive ELISA.

### Depletion and concentration of patient serum sample

Pierce^TM^ Top 12 abundant protein depletion Spin column (Thermo scientific, UK) was used to deplete OA patient serum sample as per manufacturer’s recommendations. Depleted serum was then concentrated using a freeze-dryer (MODULYOD, Thermo electron corporation). Samples were re-suspended in 50μl of deionised water and quantified using a bicinchoninic acid (BAC) protein assay kit (Fischer Scientific, UK) according to the manufacturer’s guidelines. Equal volumes (15μl) of protein sample were mixed with sample buffer (1M Tris-HCl, 10% sodium dodecyl sulfate (SDS), 40% glycerol, 0.5% Coomassie blue, and 2% 2-mercaptoethanol (Sigma, UK)). Mixed samples were boiled at 95°C for 5min and vortexed. Samples were then loaded into wells of SDS-PAGE for western-blotting.

### Western blotting

Human complement C3b (Merck, UK), human vitronectin protein (Millipore, UK), synthetic C3f peptide (Eurogentec, UK) and patient’s serum samples were separated on sodium dodecyl sulphate–polyacrylamide gel electrophoresis (SDS-PAGE) and immunoblotted with anti-C3f monoclonal antibody (mAb-C3f) and polyclonal antibodies anti-C3f and anti-V65 (pAb-C3f and pAb-V65). Blots were blocked with 5% BSA and incubated overnight at 4°C with primary antibodies. Primary antibody was revealed using an HRP-conjugated secondary antibody (GE Healthcare UK, anti-mouse IgG NA931V or anti-rabbit IgG NA934V) prior to imaging using equal volumes of luminal and peroxidase (from Super Signal West Dura, Pierce Rockford, USA) for 5min. Membrane was imaged with a ChemiDoc MP Imaging System (Bio-Rad) using Image LabTM Software version 5.0.

### Methods for enhancing assay sensitivity

Poly-streptavidin-HRP (Life Technologies, UK) was used instead of streptavidin-HRP in the standard assay protocol to enhance the sensitivity of the C3f sandwich ELISA. We then tested ELAST® (ELISA Amplification system) kit using biotinylated-Tyramide Signal Amplification (TSA) purchased from PerkinElmer, UK and used as suggested by the manufacturers. We also used dissociation-enhanced lanthanide fluorescence immunoassay (DELFIA) for enhancing the sensitivity of C3f sandwich ELISA. Briefly, the assay was performed as usual up to addition of mAb-C3f detecting antibody. After washing the plate, europium-N1-labelled anti-mouse IgG was used along with DELFIA reagents (assay buffer, enhancement solution and wash concentrate) (PerkinElmer, UK) to develop the assay as recommended by PerkinElmer. Fluorescence of each sample was read in a Wallac Victor2, 1420 multilabel counter (Perkin-Elmer, Wellesley, USA).

### Statistical analysis

Statistical analysis was performed using Graph Pad Prism software (Version 6.05). Kruskal-Wallis with post hoc Dunn’s analysis was used for multiple comparisons. Significance was considered at p < 0.05. Samples were run at least three times. Data are represented as mean ± standard error of the mean (SEM).

## Results

### Assay development

**Fig 1:**
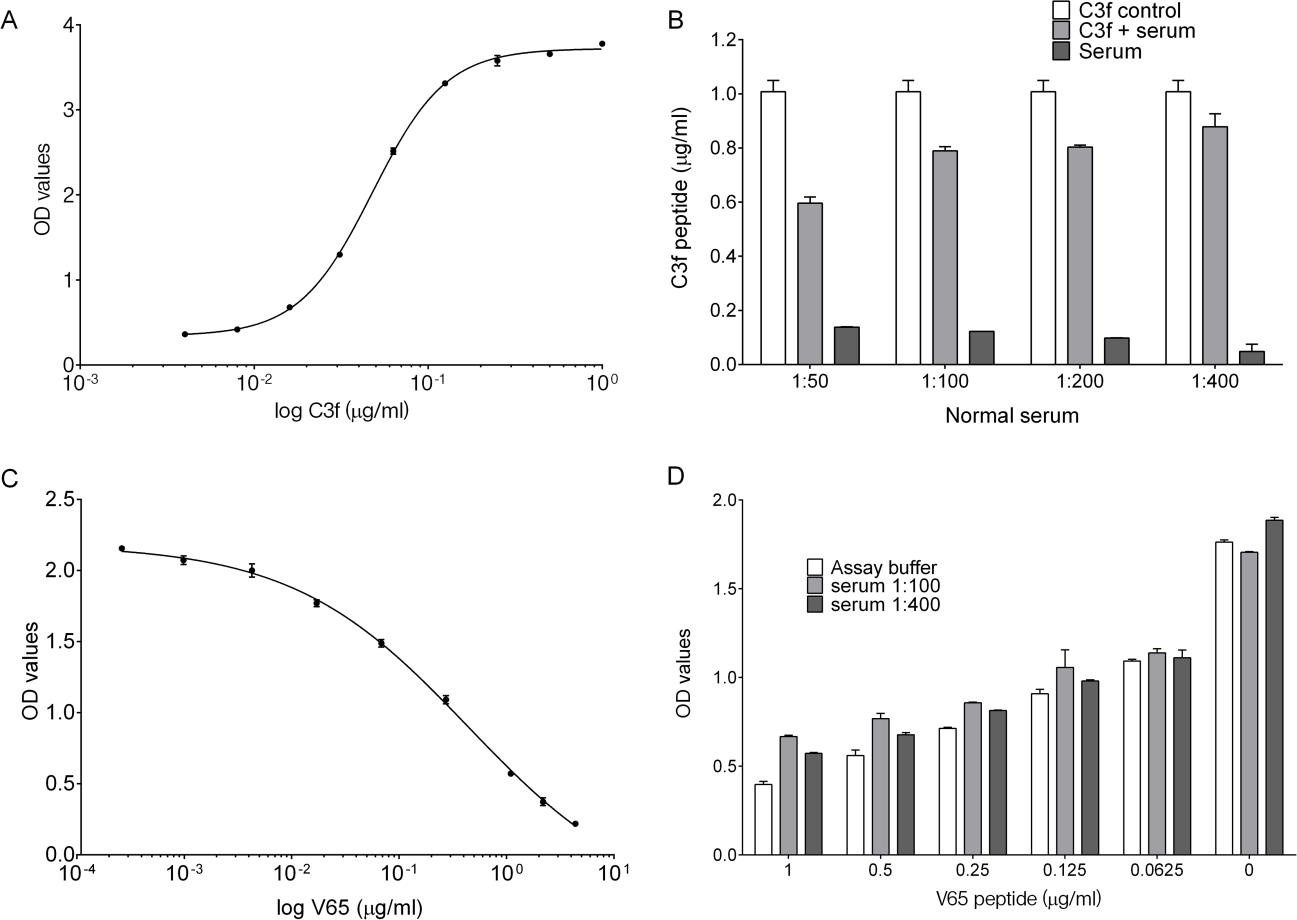
C3f and V65 ELISA development and validation. (A) A typical standard curve for C3f sandwich ELISA. Graph represent the log of C3f peptide concentration (from 0–1 μg/ml) against reading of optical density (OD) values fitted with nonlinear regression curve (Four Parameters Logistic Regression(4PL)). (B) Recovery of C3f peptide from spiked normal human serum diluted 1:50 to 1:400. C3f peptide was added at 1 μg/ml into assay buffer as positive control (C3f control) and to human serum diluted at 1:50, 1:100, 1:200 and 1:400 to test recovery. Diluted serum without spiked C3f was tested as negative control. Recovery of spiked C3f in serum was calculated in comparison to C3f control and showed 65%, 86%, 88% and 96% recovery at 1:50, 1:100, 1:200 and 1:400 dilutions respectively. Data plotted as means ± SEM. (C) A typical standard curve for V65 competitive ELISA. Graph represent the log of V65 peptide concentration (from 0–4 μg/ml) fitted with nonlinear regression curve (4PL). (D) Graph represent the concentration of V65 peptide against OD values. Spiking experiment was carried out with 2-fold serial dilution starting at 1μg/ml of V65 peptide using normal human serum (at 1:100 or 1:400). Data plotted as means ± SEM.

For C3f sandwich ELISA, a typical standard curve generated for the assay is shown in Fig 1A. The detection limit of the assay was approximately 16 ng/ml. C3f recovery rate in different serum dilution was assessed in comparison to C3f diluted in the assay buffer (Fig 1B). Using serum at 1:50, 1:100, 1:200 and 1:400 resulted in recovery of 65%, 86%, 88% and 96% of C3f peptide respectively. Lower dilutions of serum were associated with higher background signal in the ELISA (Fig 1B).

For V65 competitive ELISA, a typical standard curve generated for the assay is shown in Fig 1C. The detection limit for the V65 assay was also approximately 16 ng/ml, which represent 18% inhibition rate. Spiking test using pooled normal human serum shows that most of the spiked V65 peptide could be recovered in the dilution range 1:100 or 1:400 (Fig 1D).

### Assay variations

The intra- and inter-assay variability of the assays was checked using C3f and V65 peptides and control human serum in repeat runs of the C3f sandwich ELISA and V65 competitive ELISA. Intra- and inter-assay variations for the C3f and V65 assays were 1.3–10.8% and 4.2–10.3% respectively.

### Assay validation studies

**Fig 2:**
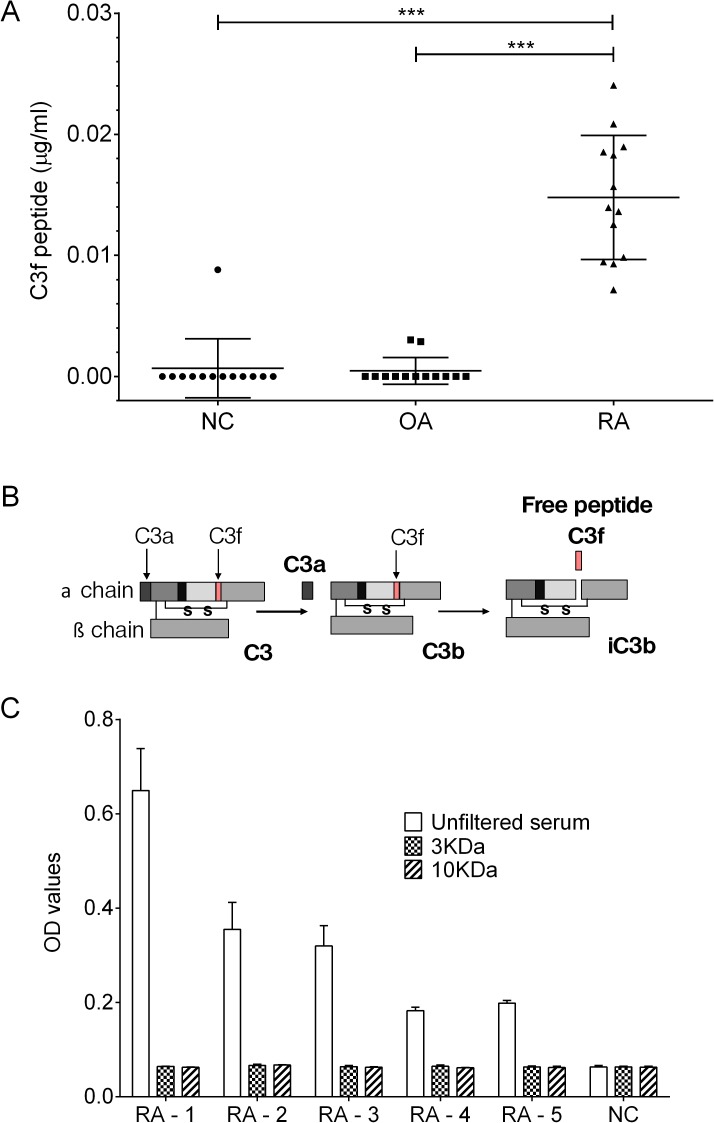
C3f in human serum samples. (A) Measurements of C3f in μg/ml in serum samples from OA patients (n = 13), RA patients (n = 13) and NC (n = 13). All samples were analysed at 1 in 50 dilution. Concentration of C3f was significantly increased in the RA group in comparison to the OA and NC group (***p>0.0001) and no increase was observed for the OA group. Data were analysed using Kruskal-Wallis with post hoc Dunn’s analysis and plotted as means ± SEM. (B) Schematic representation of human C3 complement cleavage to generated C3a, C3b, C3f and iC3b fragments. (C) C3f sandwich ELISA performed on filtered serum samples from the 3 study groups. RA patient’s sample (n = 5) and 1 NC sample were filtered using 3kDa and 10kDa cut-off filter and assessed by C3f sandwich ELISA. Unfiltered serum were tested as control. Data plotted as means ± SEM.

We analysed a sub-set of OA and control samples from the original proteomic study performed in 2011 [9] which included 13 of the OA serum samples showing highest signal in our proteomic study, 13 RA and 13 normal control. The results showed elevated C3f peptide in serum from RA patients but not in OA patients or healthy controls (Fig 2A). For V65 assay, the peptide was below the detection limit of the assay in all 3 groups (see supporting information S1 Fig).

The data in Fig 2A show that the levels of the C3f peptide in OA and healthy controls serum is very low and mainly below the detection limit of our assay. Secondly, the high C3f levels in RA serum suggest that the anti-C3f antibodies may be reacting with other larger molecules in RA serum that contain the C3f peptide. We therefore, carried out further validation studies to establish the nature of the cross-reactivity in the C3f assay.

The C3f fragment is contained within the fragment of C3 complement (C3b) as illustrated in Fig 2B. Accordingly, to demonstrate that the C3f assay is mainly detecting the C3f fragment within C3b molecule in the RA serum, 5 of the positive RA serum samples from Fig 2A above were put through 10kDa and 3kDa cut-off filter columns (Bio-Rad, UK) prior to analysis by the C3f sandwich ELISA. The data show that after filtration of the RA serum no C3f was detected by the ELISA and the OD readings for the RA group was similar to the other two groups (Fig 2C).

**Fig 3:**
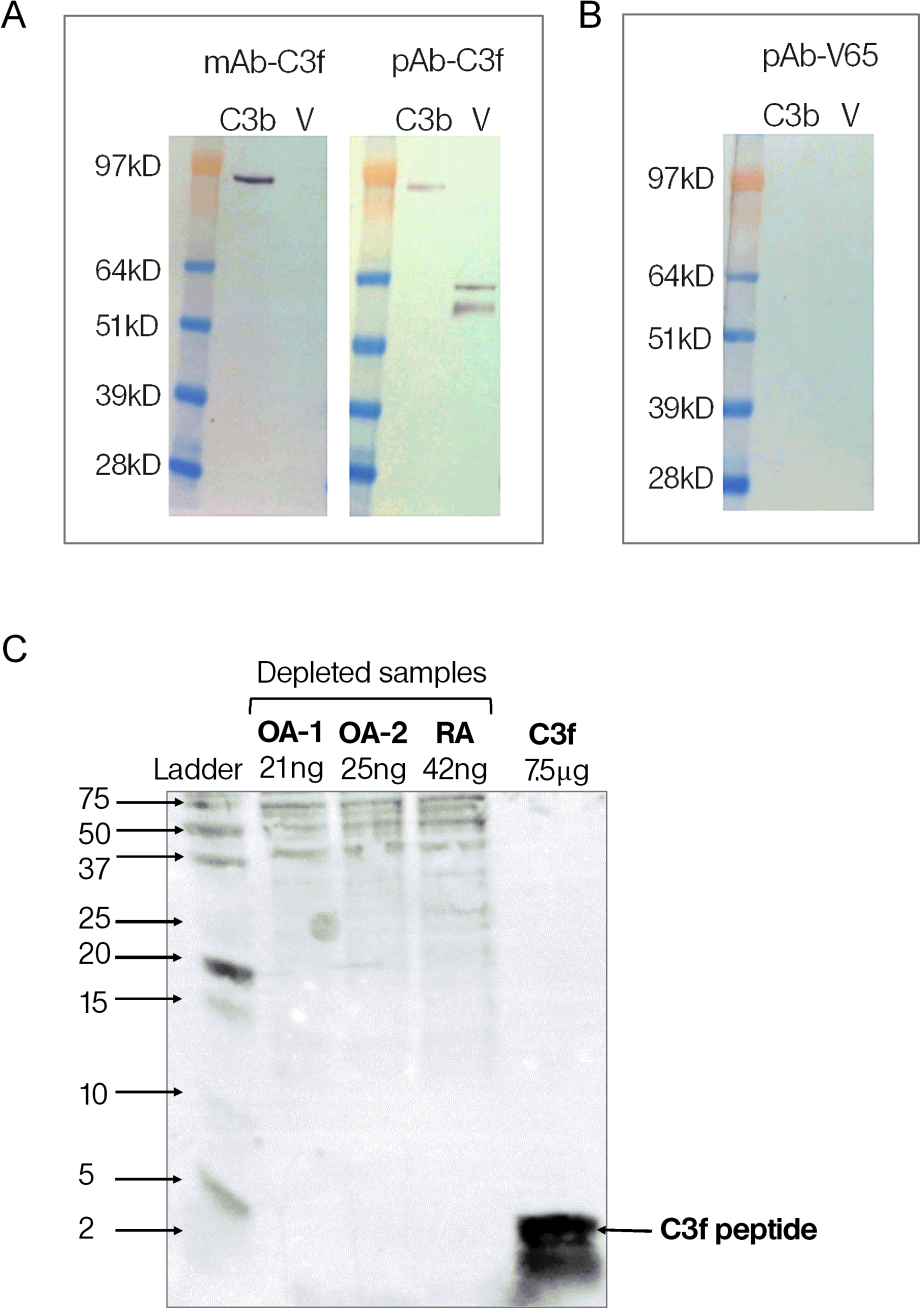
Western blot of C3f and V65. (A) Reactivity of monoclonal and polyclonal antibodies anti-C3f (mAb-C3f and pAb-C3f respectively) to C3b (Complement C3b) and human vitronectin protein (V) on western blots. (B) Reactivity of polyclonal antibodies anti-V65 (pAb-V65) to C3b and vitronectin protein on western blots. (C) Reactivity of monoclonal antibody anti-C3f on Western blots of depleted and concentrated serum samples from OA and RA patients and synthetic C3f peptide. OA sample 1 and 2 were loaded at 21ng and 25ng per lane respectively. RA and C3f samples were loaded at 42ng and 7.5μg per lane respectively.

In order to confirm the specificity and cross reactivity of the monoclonal and polyclonal antibodies, we tested the antibodies used in assays against human C3b and the vitronectin protein on western blots. The results confirmed that the two antibodies used in the C3f assay cross-react with C3b and that the polyclonal antibody (pAb-C3f) also cross-react with vitronectin protein (Fig 3A). However, the polyclonal antibody (pAb-V65) showed no cross-reactivity with either C3b or vitronectin protein (Fig 3B).

To further examine the sensitivity of the monoclonal C3f-antibody, we tested the antibody on western blots of depleted and concentrated serum from OA patients showing highest peptide levels in the proteomic study. Synthetic peptides were used as positive controls on the western blots. The antibody picked up the corresponding peptide as expected but no bands corresponding to the peptide on the depleted samples were picked up (Fig 3C).

### Approaches to improve sensitivity of the C3f and V65 assays

**Fig 4:**
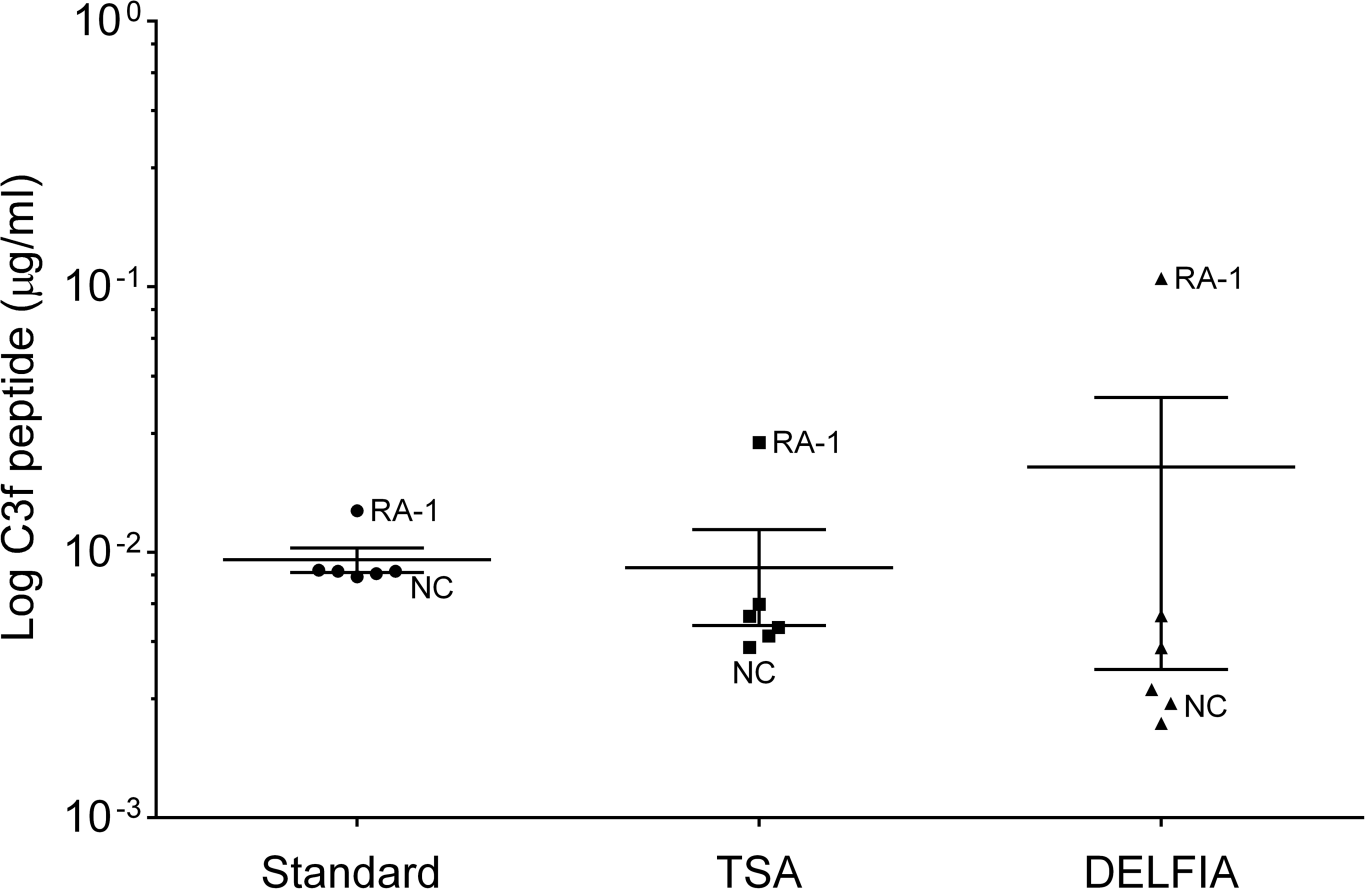
Technics to improve sensitivity of C3f and V65 assays. Graph shows the log of C3f peptide concentration detected in human serum sample using the standard sandwich ELISA (standard) and different detection system (TSA and DELFIA) for increasing sensitivity. Sample used were all from the original proteomic study: 1 NC and 1 RA and 4 OA (K&L3/K&L4). No significant change was observed between the different methods. Data were analysed using Kruskal-Wallis with post hoc Dunn’s analysis and plotted as means ± SEM.

Poly-HRP detection system was used to improve sensitivity but was not pursued as it produced rather poor and inconsistent standard curves. TSA and DELFIA^®^ detection systems were also used but did not sufficiently enhance sensitivity of the assays (Fig 4). Finally, Meso Scale technology was tested, but this platform did not generate reproducible standard curves for the assays using synthetic peptides and therefore was not pursued further.

## Discussion

We have developed two immunoassays for measurements of C3f and V65 peptides biomarkers which were discovered by our proteomic studies in 2011 [9]. Both assays show good inter and intra-assay variability and can detect the synthetic peptides in ELISA buffers and when spiked into serum samples from patients with OA and controls. The sandwich ELISA for C3f and competition ELISA for V65 detected the corresponding endogenous peptides in serum samples from patients and controls but the assays lack sensitivity and therefore could not be used for measurements of the two peptides in patients. Improving the sensitivity of the assays using different detection systems did not make any significant difference in the results.

Over the last 5 years, many groups have used proteomic studies to identify proteins that are differentially expressed in OA and therefore could be used as a biomarker for diagnosis and monitoring OA. These studies have analysed joint tissues (mainly synovium and cartilage), serum, plasma and synovial fluid from patients and controls and have identified many potential biomarkers but none of these proteins/biomarkers have turned out to be sufficiently OA–specific for development as diagnostic biomarker test for OA [11–13]. The two biomarkers that we have discovered appear to be OA-specific and showed excellent correlation with disease severity [9]. These results were recently confirmed using one of the most advanced MS-based proteomic techniques (Ion Trap) currently available [14]. Allowing for sample processing and dilution factors, the concentrations of the two biomarkers in OA serum measured by Ion Trap were in low nanogram/picogram (approximatively 1.5ng/ml) levels and therefore well below the detection limit of our ELISA assays. The first generation immunoassays for C3f and V65 reported in the current study provide a useful platform for future development of the assays as point of care diagnostic tests for OA, but further studies are required to improve the sensitivity of the assays and to investigate the possible pathological role of these peptides in OA.

The SELDI-TOF approach was used in our original MS-proteomic study for high throughput protein profiling of low molecular weight peptides/proteins (below 20 kDa). Such small fragments pose significant technical challenges for raising suitable high affinity antibodies and development of sensitive immunoassays. For antibody production, we had to ensure that there were no common linkers in the peptides and that the response is truly due to peptide recognition. Therefore, a number of different coupling chemistries were tested to select the best one for the preparation of the immunogen linking to carrier proteins KLH or BSA. Secondly, we had to immunise at least 3 rabbits and 6 mice with each of the peptides to obtain a reasonable immune response to the peptides in one rabbit and one mouse.

A further technical problem we encountered during assay validation studies was that the monoclonal and polyclonal antibodies to C3f used in the C3f assay cross-reacted with C3b and the human vitronectin protein respectively. Both C3f and V65 are small peptides of ~2kDa and larger fragments containing these peptides are known to exist in the blood [12]. For example, C3f is a fragment released during the catabolic degradation of C3b complement and since RA is a highly inflammatory disease, the presence of a large amount of complement in the patient blood may account for the increased levels of C3f seen in the RA samples in Fig 2A.

Our study highlights the difficulties and challenges of validating novel biomarker from proteomic studies for use in patients. In this study, we have demonstrated how to overcome some of the technical challenges associated with developing immunoassays for small peptides from proteomic studies, but as MS offers almost infinite sensitivity, how to achieve such high sensitivity in simple immunoassays remains a major issue. There are several ways to make the assays super sensitive but these would require the use of completely different platform and generation of new high affinity antibodies. One possibility would be to use the Human Combinatorial Antibody Library (HuCAL^®^) technology. This is a powerful technique that allows for selection of high affinity antibodies *in vitro* without having to immunise a large number of animals to find the best responder to the antigen [15]. Production of monoclonal antibodies by HuCAL technology is likely to help develop more robust and sensitive ELISAs for quantitative measures of the two novel biomarkers in patients. This is clearly beyond the scope of the current study.

OA is historically considered a non-inflammatory disease but the role of pro-inflammatory cytokines and other mediators of joint inflammation in both initiation and progression of joint damage in OA is now well established [16]. Both C3f and V65 may be associated with inflammatory responses in OA. For example, cartilage extracellular matrix contains small leucine-rich repeat proteins some of which are thought to interact with the globular head domain of C1q to further activate the classical and alternative pathways of complement factors [17]. Similarly, vitronectin protein is recognised by αVβ3 integrin receptor and interaction between vitronectin via its arginine glycine and aspartic acid motif may be involved in regulation of inflammatory mediators (IL-1β, NO and PGE_2_) in OA cartilage [18]. Accordingly, in parallel to studies to develop sensitive assays for the two novel biomarkers our future studies will explore the physiological and pathological functions of C3f and V65 peptides.

## Conclusion

We have developed immunoassays for measurements of C3f and V65 peptides which were identified as potential OA–specific biomarkers by our MS-based proteomic studies. These assays can detect the endogenous peptides in serum samples from patients and controls but lack sensitivity for accurate measurements of the peptides in patients. Therefore, the development of more robust and highly sensitive immunoassays is required to confirm the proteomic study findings and to qualify these two novel biomarkers as diagnostic and/or prognostic markers of OA.

## Supporting information

S1 Fig V65 in human serum sample. V65 in serum samples from OA (n = 24), RA (n = 5) and normal matched controls (NC, n = 5). All samples were analysed at 1 in 50 dilutions. Data are plotted as % inhibition = 100- ((Mean absorbance of test sample/Mean absorbance anti-V65 control) x 100). No significant change was observed between the different groups. Data were considered negative as they all are below 20% inhibition. Data were analysed using Kruskal-Wallis with post hoc Dunn’s analysis and plotted as means ± SEM.
(TIF)

S2 Fig Primary data for Fig 2A and 2D. **2A:**C3f concentration in μg/ml in serum samples from Osteoarthritis (OA) (n = 13), Rheumatoid arthritis (RA) (n = 13) and normal control (NC) subjects (n = 13) used in Fig 2A. **2C**: OD values after C3f sandwich ELISA performed on filtered serum samples from OA, RA and NC subjects used in Fig 2C.
(DOCX)

S3 Fig Primary data for Fig 4. Estimated concentration of C3f in μg/ml in serum samples which were below the detection limit for all three assays.
(DOCX)
