# Supplementary material for: Development and validation of novel biomarker assays for osteoarthritis
Source: PLoS One. 2017 Jul 17;12(7):e0181334. doi: 10.1371/journal.pone.0181334 (PMC5513499; doi:10.1371/journal.pone.0181334)
Supplement: S2 Fig — 2A:C3f concentration in μg/ml in serum samples from Osteoarthritis (OA) (n = 13), Rheumatoid arthritis (RA) (n = 13) and normal control (NC) subjects (n = 13) used in Fig 2A. 2C: OD values after C3f sandwich ELISA performed on filtered serum samples from OA, RA and NC subjects used in Fig 2C. (DOCX) [file pone.0181334.s002.docx]

**ON-LINE supplement**

**Development and validation of novel biomarker assays for osteoarthritis**

Khadija Ourradi^1^, Yunhe Xu^1^, Dominique de Seny^2^, John Kirwan^3^, Ashley Blom^1^ and Mohammed Sharif^1*^

**S2 - Fig**

**2A**

| **C3f [µg/ml]** | | |
| --- | --- | --- |
| **NC** | **OA** | **RA** |
| 0.000000 | 0.000000 | 0.009302217 |
| 0.008807122 | 0.000000 | 0.00983928 |
| 0.000000 | 0.000000 | 0.01255231 |
| 0.000000 | 0.003026646 | 0.007158821 |
| 0.000000 | 0.002862743 | 0.01827643 |
| 0.000000 | 0.000000 | 0.01362105 |
| 0.000000 | 0.000000 | 0.02404491 |
| 0.000000 | 0.000000 | 0.013947 |
| 0.000000 | 0.000000 | 0.01853236 |
| 0.000000 | 0.000000 | 0.02084998 |
| 0.000000 | 0.000000 | 0.01895734 |
| 0.000000 | 0.000000 | 0.00946268 |
| 0.000000 | 0.000000 | 0.01567428 |

**2C-**

|  | **OD values** | | | | | | | | |
| --- | --- | --- | --- | --- | --- | --- | --- | --- | --- |
| **Samples** | **Unfiltered serum** | | | **3KDa** | | | **10KDa** | | |
| **RA - 1** | 0.7739 | 0.6975 | 0.4770 | 0.0647 | 0.0633 | 0.0650 | 0.0642 | 0.0611 | 0.0630 |
| **RA - 2** | 0.4691 | 0.3051 | 0.2910 | 0.0621 | 0.0665 | 0.0710 | 0.0657 | 0.0677 | 0.0690 |
| **RA - 3** | 0.4047 | 0.268 | 0.2870 | 0.0633 | 0.0601 | 0.0690 | 0.06 | 0.0645 | 0.0640 |
| **RA - 4** | 0.1973 | 0.1778 | 0.1730 | 0.061 | 0.0635 | 0.0700 | 0.0614 | 0.0603 | 0.0630 |
| **RA - 5** | 0.2107 | 0.1927 | 0.1920 | 0.0658 | 0.06 | 0.0650 | 0.0577 | 0.0618 | 0.0670 |
| **NC** | 0.0606 | 0.0615 | 0.0690 | 0.0603 | 0.0652 | 0.0650 | 0.06 | 0.0621 | 0.0670 |
