# Supplementary material for: Development and validation of novel biomarker assays for osteoarthritis
Source: PLoS One. 2017 Jul 17;12(7):e0181334. doi: 10.1371/journal.pone.0181334 (PMC5513499; doi:10.1371/journal.pone.0181334)
Supplement: S3 Fig — Estimated concentration of C3f in μg/ml in serum samples which were below the detection limit for all three assays. (DOCX) [file pone.0181334.s003.docx]

**ON-LINE supplement**

**Development and validation of novel biomarker assays for osteoarthritis**

Khadija Ourradi^1^, Yunhe Xu^1^, Dominique de Seny^2^, John Kirwan^3^, Ashley Blom^1^ and Mohammed Sharif^1*^

**S3 Fig**

|  | **C3f [µg/ml]** | | |
| --- | --- | --- | --- |
| **Samples** | **Standard** | **TSA** | **DELFIA** |
| **OA-1** | 0.008476 | 0.005212 | 0.004367638 |
| **OA-3** | 0.008306 | 0.004842 | 0.003038205 |
| **OA-4** | 0.008491 | 0.005740 | 0.002265791 |
| **OA-5** | 0.008563 | 0.006375 | 0.005763505 |
| **RA-1** | 0.014319 | 0.025871 | 0.1074546 |
| **NC** | 0.008077 | 0.004386 | 0.002698338 |
